# Efficacy and Optimal Pressure of Continuous Positive Airway Pressure in Intensity-Modulated Radiotherapy for Locally Advanced Lung Cancer

**DOI:** 10.3390/cancers14174308

**Published:** 2022-09-02

**Authors:** Jaehyeon Park, Ji Woon Yea, Se An Oh, Jongmoo Park, Jae Won Park, Jeong Eun Lee

**Affiliations:** 1 Department of Radiation Oncology, Yeungnam University Medical Center, Daegu 42415, Korea; 2 Department of Radiation Oncology, Yeungnam University College of Medicine, Daegu 42415, Korea; 3 Department of Radiation Oncology, School of Medicine, Kyungpook National University, Daegu 41944, Korea

**Keywords:** radiotherapy, lung cancer, motion management, continuously positive airway pressure

## Abstract

**Simple Summary:**

Radiation pneumonitis is a major late complication in radiotherapy (RT) for lung cancer. Respiratory gating radiotherapy and deep inspiration breath hold are representative techniques to protect the normal lung by managing the movement of the tumor. However, these are highly patient-dependent techniques. Continuous positive airway pressure (CPAP) is used as an alternative, but it is unclear how much pressure will be effective. We aimed to determine the optimal pressure of CPAP for RT through changes in the dosimetric parameters and lung volume according to pressure. The air pressure was raised in five steps of 4, 7, 10, 14, and 17 cmH_2_O and a CT scan was performed at the baseline and at each pressure step, accompanied by contouring and RT planning. CPAP linearly increased lung volume and decreased the dosimetric parameter in the pressure range 7 to 13 cmH_2_O (*p* < 0.01). Above 13 cmH_2_O, V5 of the heart also showed a significant decrease (*p* < 0.01).

**Abstract:**

We aimed to determine the optimal pressure of continuous positive airway pressure (CPAP) for radiotherapy (RT) through changes in the dosimetric parameters and lung volume according to pressure. Patients with locally advanced lung cancer, who underwent CPAP during computed tomography (CT) simulation, were included. The air pressure was raised in five steps of 4, 7, 10, 14, and 17 cmH_2_O and a CT scan was performed at the baseline and at each pressure step, accompanied by contouring and RT planning. Paired t- and Wilcoxon signed rank tests were used to compare the volumetric and dosimetric parameters according to pressure and interpressure. A total of 29 patients were selected, and 158 CT datasets were obtained. The lung volume increased significantly at all pressures (*p* < 0.01). The Dmean of the lung decreased significantly from 7 cmH_2_O (*p* < 0.01), the V5, V10, V15, and V20 of the lung decreased significantly from 7 cmH_2_O with increasing pressure, and the Dmean and V5 of the heart decreased significantly from 14 cmH_2_O with increasing pressure. The V50 showed no significant differences at any pressure. We recommend the use of at least 7 cmH_2_O with 14 cmH_2_O as the optimal pressure to achieve the effect of heart preservation.

## 1. Introduction

Lung cancer is the most commonly diagnosed cancer and the leading cause of cancer-related deaths worldwide [1]. Radiotherapy (RT) is an important modality in the treatment of lung cancer, such as stereotactic body radiotherapy (SBRT) for inoperable early-stage non-small cell lung cancer (NSCLC) and concurrent chemoradiotherapy (CCRT) for locally advanced NSCLC or limited-disease small cell lung cancer (LD-SCLC).

Radiation pneumonitis (RP) is a major late complication and an obstacle to treatment. As a result, interest in treatment-related pneumonitis is increasing. Symptomatic RP occurs in 15–40% of patients receiving CCRT for NSCLC [2,3]. In particular, the use of durvalumab, an anti-programmed death ligament 1 (PD-L1) inhibitor after CCRT, has become the standard treatment for unresected stage III NSCLC. Some prospective studies have reported increased pulmonary toxicity in patients who received RT with PD-1 or PD-L1 inhibitors [4,5].

Many efforts have been made to identify the predictive factors related to the incidence of RP. The Quantitative Analysis of Normal Tissue Effect in Clinic (QUANTEC) project reviewed more than 70 published articles and presented normal tissue constraints for radiation-induced lung injury [6]. According to this project, dosimetric parameters for the risk of RP <20% were suggested as mean lung dose (MLD) <20 Gy, V20 (the percentage of the lung volume receiving at least 20 Gy) <35%, and V5 (the percentage of the lung volume receiving at least 5 Gy) <60% in conventional fractionation.

The lung is an organ that expands and contracts repeatedly with respiration, and a lung cancer tumor that is located within it also moves with respiration. Intra-fractional respiratory-related motion causes uncertainties in the target coverage and dose delivery. To cover these movements, the treatment volume is increased, with the volume of the normal lung irradiated also being increased accordingly. Respiratory gating radiotherapy (RGRT) and deep inspiration breath hold (DIBH) are representative techniques for reducing the dose irradiated to the normal lung by managing the movement of the tumor. However, there are several conditions for using these techniques. Irregular breathing causes poor dosimetric results in the RGRT. Therefore, a regular respiratory cycle is required for the stability and reproducibility of the treatment. DIBH also requires the same reproducible inspiration level during the simulation and treatment. In other words, both techniques are highly patient-dependent and require patient cooperation and education for proper treatment.

Continuous positive airway pressure (CPAP) is a form of positive airway pressure ventilation that delivers a constant level of pressure to the airway to prevent airway collapse. It has been used as a safe and effective treatment for patients with sleep apnea [7,8], and has recently been used for chronic obstructive pulmonary disease (COPD) [9] and to prevent postoperative atelectasis [10]. CPAP causes the airway to be continuously open and, consequently, lung inflated compared to free breathing. Several attempts have been made to use this effect for RT, which were mainly performed in patients with left breast cancer and lymphoma who were unable to undergo DIBH [11,12]. In a study of patients with left breast cancer, CPAP increased the lung volume by 35% and decreased the mean dose of the heart and ipsilateral lung and V20 of the lung by 47%, 20%, and 25%, respectively [13].

Most previous studies have focused on patients with left breast cancer, and there are no studies on the dosimetric improvement of the CPAP application in RT for locally advanced lung cancer. In addition, there are no studies on how much pressure has a meaningful effect on the dosimetric outcomes. Therefore, we conducted a study to determine the optimal pressure of CPAP for RT through changes in the dosimetric parameters and lung volume according to pressure.

## 2. Materials and Methods

### 2.1. Patients

**Figure 1:**
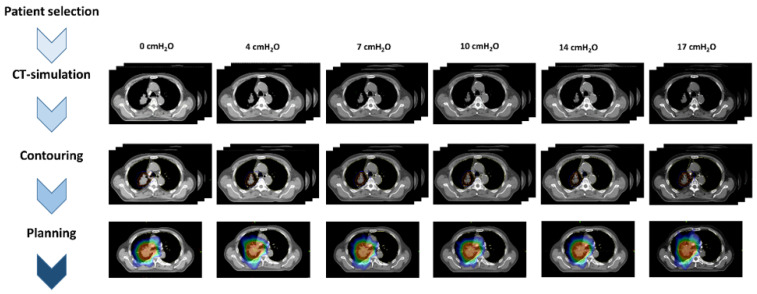
Study workflow chart.

This study was approved by the Institutional Review Board (2021-08-058). The progress of this study is summarized in Figure 1. We selected patients with locally advanced lung cancer who underwent CPAP during computed tomography (CT) simulation between July 2019 and December 2020. A total of 29 patients were included in the study. Table 1 shows the patient characteristics. The median age was 67 years (range, 51–82). There were 22 patients with NSCLC (76%) and 7 patients with SCLC (24%). Most tumors (59%) were stage IIIA according to the American Joint Committee on Cancer (AJCC) 8th edition. The most common lesions were in the upper lobe; 14 patients had lesions in the RUL, and 7 in the LUL. Thirteen patients (44%) had respiratory comorbidities.

### 2.2. Application of CPAP on CT-Simulation

We used AirSense^TM^10 (ResMed, San Diego, CA, USA) as the CPAP machine, and all the patients wore CPAP masks that fitted their face sizes. These were worn for 5–10 min at a pressure of 4 cmH_2_O for adaptation to the CPAP before the CT simulation. All patients were simulated in the supine position with the arm up and immobilized with a Vac-lok bag and wing board. The air pressure was raised step-by-step to the patient’s individual tolerable pressure in five steps of 4, 7, 10, 14, and 17 cmH_2_O. We used the Brilliance Big Bore CT simulator (Philips Inc., Cleveland, OH) and a CT scan with a 2.5-mm slice thickness was performed at the baseline and each pressure step.

### 2.3. Contouring and RT Planning

All CT datasets were transferred to a commercial treatment planning system (Varian Eclipse TPS version 15.6.05, Palo Alto, CA, USA). The delineation of the target volume and normal organs, including the esophagus and heart, was conducted by an experienced radiation oncologist (J.P.). The lung contouring was performed using auto-segmentation of the TPS. The gross target volume (GTV) was defined as primary lesions and lymph nodes that showed positive findings on the positron emission tomography-CT and contrast-enhanced chest CT. This was expanded with a 5-mm margin to generate the clinical target volume (CTV). The planning target volume (PTV) was obtained by adding a craniocaudal 7-mm and radial 5-mm margin to the CTV.

A treatment plan was generated for each CT set using an anisotropic analytic algorithm. The prescribed radiation dose was 66 Gy in 33 fractions with a fraction size of 2 Gy delivered to the PTV, with 95% of the PTV being covered by the prescription dose. The dose constraints for the organs at risk (OAR) are summarized in Table S1. Volumetric modulated arc therapy was used in 27 patients, and nine-field static intensity-modulated radiotherapy was used in 2 patients.

### 2.4. Statistics

Paired t- and Wilcoxon signed rank tests were used to measure meaningful changes in the dosimetric outcomes, including lung volumes, lung-dose parameters (D_mean_, V5, V10, V15, and V20), and heart dose parameters (D_mean_, V5, and V50).

The difference in the parameters according to pressure was defined as follows:

$$\mathrm{Relative}\;\mathrm{change}\;\left(\%\right)=\frac{P_{x}-P_{0}}{P_{0}}\times 100$$

where *P_x_* was the parameter in pressure *x*, and *P*_0_ the parameter at baseline. The difference in the parameter between the interpressure was defined as follows:

$$\mathrm{Relative}\;\mathrm{change}\;\left(\%\right)=\frac{P_{x+1}-P_{x}}{P_{x}}\times 100$$

where *P_x_*_+1_ was a parameter in pressure step *x* + 1 and *P_x_* a parameter in pressure *x*. All the statistical analyses were performed using SPSS ver. 25.0 (SPSS Inc., Chicago, IL, USA). 

## 3. Results

**Figure 2:**
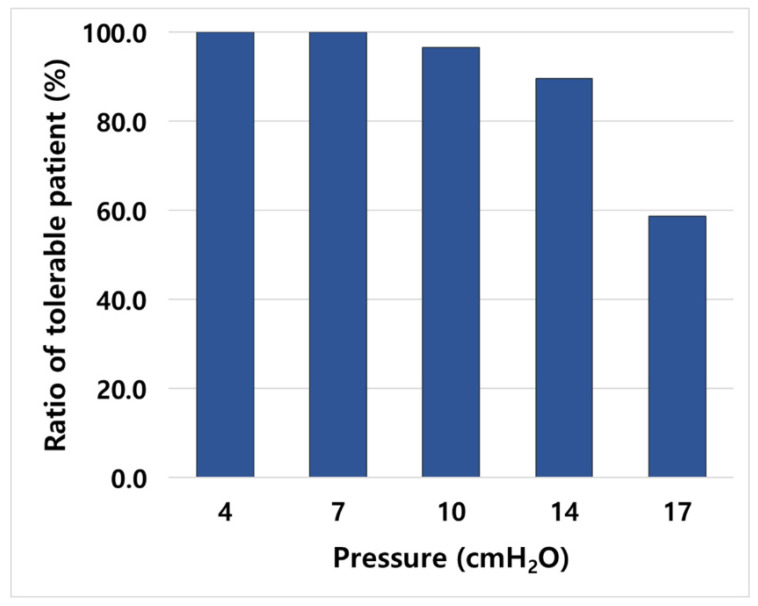
Cumulative proportion of tolerable patients at each pressure.

A total of 158 CT datasets were obtained with 1, 2, 9, and 17 patients who tolerated up to 7, 10, 14, and 17 cmH_2_O, respectively. Cumulatively, approximately 90% of the patients tolerated up to 14 cmH_2_O, and only 59% of the patients tolerated up to 17 cmH_2_O (Figure 2).

The volume of GTV, CTV and PTV at baseline were 77.3 ± 48.4 cm^3^, 161.7 ± 78.3 cm^3^ and 292.3 ± 129.0 cm^3^, respectively. There were no statistically significant differences in the target volumes according to the pressure (Table 2).

### 3.1. Change in Lung Volume and Dose Parameters

**Figure 3:**
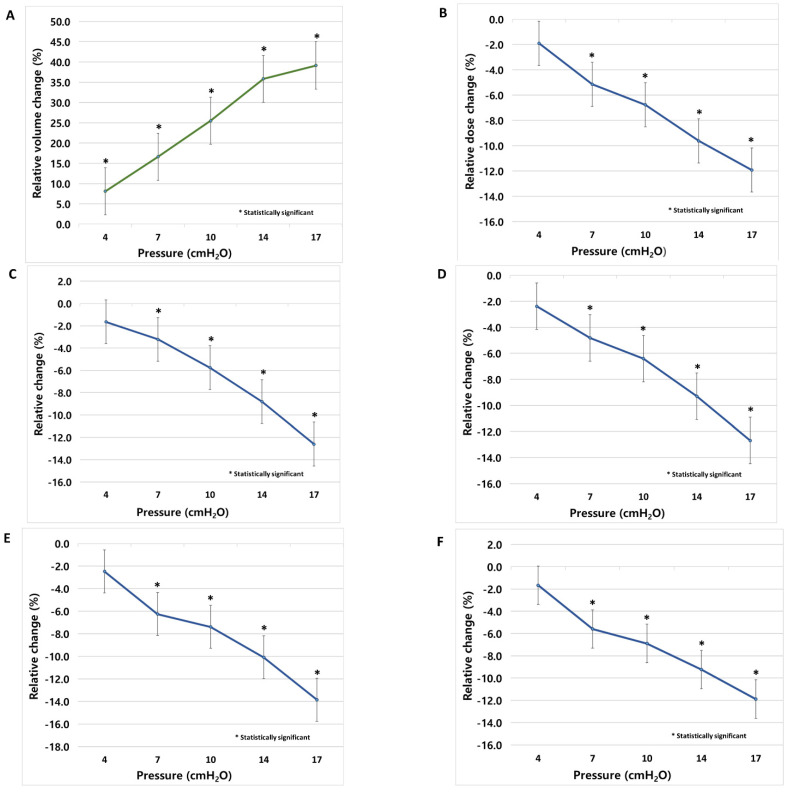
Lung volume and dose parameter change comparison with baseline; (**A**) volume, (**B**) D_mean_, (**C**) V5, (**D**) V10, (**E**) V15, and (**F**) V20. Statistically significant, *.

The volumes of the lung were 3318.0 ± 742.4 cm^3^, 3575.4 ± 762.1 cm^3^, 3840.1 ± 764.5 cm^3^, 4148.9 ± 820.5 cm^3^, 44529.5 ± 905.9 cm^3^ and 4883.2 ± 799.4 cm^3^ from baseline in the order of 4, 7, 10, 14 and 17 cmH_2_O (Table 2). The lung volume increased significantly at all pressures, and the increase was linear (Figure 3A). It increased by approximately 8% with each increase in pressure step; however, the degree of increase decreased to 3% when increasing from 14 to 17 cmH_2_O (Figure S1(A1)).

The D_mean_ for the lung were 1573.5 ± 314.0 cGy, 1545.9 ± 327.7 cGy, 1495.3 ± 330.8 cGy, 1455.9 ± 334.6 cGy, 1392.8 ± 304.4 cGy and 1443.4 ± 268.7 cGy from baseline in the order of 4, 7, 10, 14 and 17 cmH_2_O (Table 2). It decreased significantly from 7 cmH_2_O and decreased by 5.1–11.9% as the pressure increased (Figure 3B). Significant decreases were observed when the interpressure was increased from 4 to 7 cmH_2_O and from 10 to 14 cmH_2_O (Figure S1(A2)).

V5, V10, V15, and V20 also showed a significant decrease from 7 cmH_2_O with decreases of 3.2–12.6%, 4.8–12.7%, 6.3–13.8%, and 5.6–11.9%, respectively, with increasing pressure (Figure 3C–F). With the change in the interpressure, V5 decreased significantly only when increasing from 10 to 14 cmH_2_O, and V10, V15, and V20 decreased significantly only when increasing from 4 to 7 cmH_2_O (Figure S1(A3–A6)).

### 3.2. Change in Heart Volume and Dose Parameters

The volumes of the heart were 725.6 ± 155.4 cm^3^, 719.3 ± 162.0 cm^3^, 702.6 ± 167.5 cm^3^, 683.8 ± 134.5 cm^3^, 679.0 ± 109.3 cm^3^, and 622.8 ± 102.4 cm^3^ from baseline in the order of 4, 7, 10, 14, and 17 cmH_2_O (Table 2). The heart volume decreased significantly at 14 and 17 cmH_2_O. 

**Figure 4:**
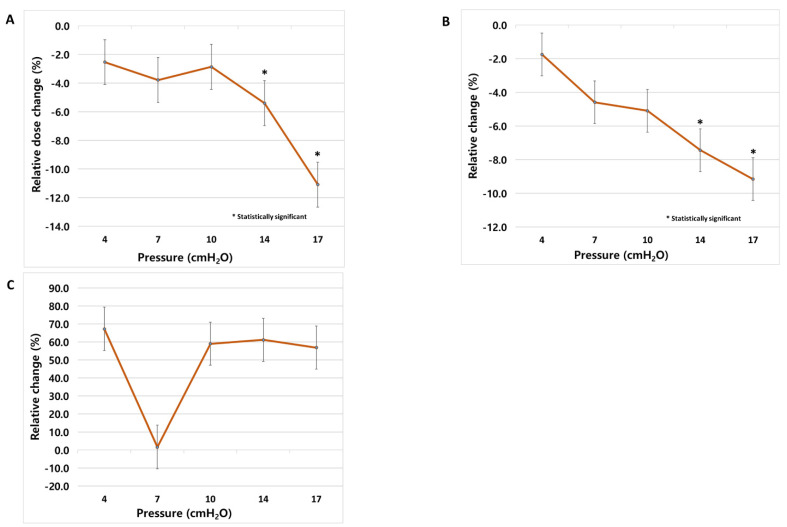
Heart dose parameter change; comparison with baseline; (**A**) D_mean_, (**B**) V5, and (**C**) V50.

D_mean_ and V5 showed significant decreases from 14 cmH_2_O, with decreases of 5.4–11.1% and 7.4–9.2%, respectively, with increasing pressure (Figure 4A,B). V50 showed no significant differences at any pressure (Figure 4C). In the analysis of the interpressure, there were no intervals showing significant differences in D_mean_, V5, or V50 (Figure S2(B1–B3)).

## 4. Discussion

In this study, changes in the lung volumes and dosimetric parameters according to pressure were confirmed using conventional RT for lung cancer. To control the effect of the target volume on the RT plan, one radiation oncologist performed the contouring, and there were no significant differences in the pressure. The lung volume increased linearly with pressure, and the dose parameters, including D_mean_, V5, V10, V15, and V20, showed a linear tendency to decrease. While the change in lung volumes was significant at all pressures, all the dose parameters showed a significant change from 7 cmH_2_O. The volume increase in the interpressure was approximately 8%, but when the pressure was increased from 14 to 17 cmH_2_O, the increase decreased to 3%. In addition, the proportion of tolerable patients was reduced from 90% to 59%. Based on these results, we suggest that in conventional RT for lung cancer, an appropriate pressure range is 7–14 cmH_2_O.

The incidence of RP was reported to be 9.1% higher with the combination of durvalumab and CCRT compared to CCRT alone in the PACIFIC trial [14], raising interest in RP in conventional RT for lung cancer. Wang et al. investigated the factors associated with RP in NSCLC patients after CCRT [15]. Lung volume, mean lung dose, V5 through V65, and target volume were all significant factors for RP in the univariate analysis, but only V5 was significant in the multivariate analysis. The 1-year incidence of grade 3 ≥ RP with V5 ≤ 42% and V5 > 42% was 3% and 38%, respectively (*p* < 0.01). Palma et al. also reported predictive factors of RP in patients undergoing CCRT for NSCLC through an individual patient data meta-analysis [3]. They suggested that the V20 of the lung, carboplatin/paclitaxel chemotherapy, and age were predictive factors.

Since the lung cancer tumor is located in the lungs and it moves according to respiration, it is necessary to manage its movement to reduce the irradiation volume of normal lungs. RGRT is commonly used for motion management. RGRT is a method of irradiating only a specific respiratory window, so the target volume can be reduced compared to free breathing [16]. Rouabhi et al. reported that RGRT could reduce the mean lung dose and V20 by 6–16% and 7–20%, respectively, compared to the non-gated plans [17]. However, this method requires patient collaboration and a regular breathing pattern. Irregular breathing causes geometric variations, which have a significant impact on the accuracy of target contouring and treatment [18]. In addition, end-expiration is preferred to end-inspiration for the gating window because of the stability and smallness of tumor movement [19]. Expiration is the densest state of the lungs and the benefit of RGRT may be dampened, due to the large amount of lung parenchyma per volume.

In contrast, DIBH, another commonly used technique, is a method of sparing the lungs using lung expansion at deep inspiration and tumor immobilization by breath-holding. This method has been studied mainly in breast cancer to reduce the heart dose [20,21,22]. Swanson et al. reported that DIBH also reduced ipsilateral lung parameters, such as mean lung dose, V5, V10, V15, and V20 by 5–16%, significantly [21]. The efficacy of DIBH has also been confirmed in lung cancer. Rosenzweig et al. showed that the normal tissue complication probability (NTCP) of radiation pneumonitis decreased in patients with DIBH, compared to patients with free breathing, in conventional RT for NSCLC [23]. Hanley et al. reported high reproducibility of DIBH with 1 mm and 2.5 mm in the deviation of intra breath-hold and inter breath-hold, respectively, as well as improved dose parameters of lung in conventional RT for lung cancer [24]. However, patient cooperation is essential for stable and reproducible treatment. In the INHALE trial, 28% of the patients were unable to perform DIBH throughout the treatment course [25].

Most of the patients enrolled in our study were elderly, and 44% of patients tolerated CPAP well, even though they had respiratory comorbidities. CPAP supplies positive pressure to keep the airways open, allowing the lungs to be inflated continuously. Thus, a similar effect to DIBH can be expected. Kil et al. used CPAP in patients with breast cancer who had difficulty in performing DIBH [12,13]. A pressure of 8–15 cmH_2_O was used. Compared with free breathing, the total lung volume was increased by 30% (*p* < 0.01), and the D_mean_ and V20 of the ipsilateral lung decreased by 20% and 25%, respectively (*p* = 0.03, both). The D_mean_ and V25 of the heart also decreased by 47% and 87%, respectively (*p* < 0.01).

Another advantage of using CPAP in RT is that it is economical. In using CPAP in RT, no special equipment is required, except for the CPAP machine and a mask for each patient. The CPAP machine we used was approximately USD 1500 and a mask was USD 250, which is a very low cost compared to a real-time position management system or the surface guided radiation therapy for DIBH or RGRT, which costs hundreds of thousands of dollars to equip. Therefore, it can be used as an effective method for protecting the lung and heart in radiation oncology departments in a financially constrained environment.

Goldstein et al. conducted a controlled pilot study to confirm the effect of CPAP on SBRT [26]. They used a pressure of 10–15 cmH_2_O, and most of the patients tolerated CPAP. CPAP significantly decreased tumor movement in all directions (superior–inferior, 0.5 ± 0.8 cm; right–left, 0.4 ± 0.7 cm; anterior–posterior, 0.6 ± 0.8 cm). A relative increase of 32% in lung volume was observed. Our study showed that the relative increases in the lungs at 10 and 14 cmH_2_O were 26% and 36%, respectively, considering the pressure range they used, and the degree of lung expansion appeared to be consistent. The D_mean_ of the lung and heart also showed relative reductions of 22% and 29%, respectively, which were larger than those of approximately 12% and 11% at 17 cmH_2_O in our study. This difference was due to the large target volume because our study investigated conventional RT for stages II-III.

The heart-sparing effect of CPAP was also observed in the present study. The dosimetric parameters of the heart are known to be associated with lymphopenia, which is an important prognostic factor in lung cancer [27]. The mean dose and low-dose exposure area of the heart also decreased significantly at 14 cmH_2_O or higher. However, V50, a high-dose exposure area of the heart, did not show significant improvement even at high pressure. This result is presumably because most of the patients included in this study had upper lobe lesions.

Recently, Wiezman et al. evaluated the volume, position, and motion of the heart at free-breathing and 15 cmH_2_O to determine the mechanism of dosimetric improvement from CPAP [28]. They reported a 6% reduction in heart volume (*p* < 0.008), 1 cm of caudal displacement (*p* < 0.008), and a 49% reduction in movement (*p* < 0.01) with CPAP. Among them, the caudal displacement of the heart was correlated with increased lung volume. In our study, there was also a significant decrease in heart volume at pressures above 14 cmH_2_O. We did not expect a significant change in the heart volume by the airway pressure, since it depends on the blood volume according to the cardiac cycle. It may have been caused by a reduction in the amount of blood flow into the heart, due to an increase in intrathoracic pressure. 

There were some limitations to this study. First, the selection of the tumor location was biased. Since the patients were selected retrospectively, 76% of the patients had tumors in the upper lobe. Therefore, we were unable to analyze how the effects of CPAP differed according to tumor location. Second, four-dimensional CT (4D-CT) was not used for the CT simulation. Four-dimensional CT can determine the extent of tumor movement during respiration. The degree of reduction in tumor movement according to pressure makes it possible to set the PTV margin for each pressure, which can potentially further reduce the irradiation dose to normal organs, such as the lungs and heart. Third, this study focused on the changes in lung volume and dosimetric parameters according to pressure in the simulation. It was not clear whether the lung volume increased, similar to the simulation, when the same pressure is applied during treatment. For practical application, further studies are needed to confirm whether the lung volume increases consistently when the same pressure is applied during treatment.

## 5. Conclusions

This is the first study to demonstrate the relationship of pressure with lung volume and dosimetric parameters. Considering the increase in lung volume, the decrease in the dosimetric parameters, the proportion of tolerable patients, and the change in lung volume between pressures, we recommend using at least 7 cmH_2_O. In conclusion, a pressure of 14 cmH_2_O, which can even achieve the effect of preserving the heart, can be considered optimal.

## Figures and Tables

**Table 1:** Summary of patient characteristics.

Variable	No. of Patients
Age (yr)	
Median (range)	67 (51–82)
Gender	
Male	28
Female	1
Histology	
Non-small cell lung cancer	22
Squamous cell carcinoma	15
Adenocarcinoma	5
NOS	2
Small cell lung cancer	7
Staging (AJCC 8th edition)	
IIB	3
IIIA	17
IIIB	6
IIIC	3
Tumor location	
Right upper lobe	14
Right middle lobe	1
Right lower lobe	5
Left upper lobe	7
Left lower lobe	1
Both upper lobe	1
Respiratory comorbidity	
Chronic obstructive pulmonary disease	11
Emphysema	1
Idiopathic pulmonary fibrosis	1

**Table 2:** Comparison of structure volume and dosimetric parameters at each pressure.

Structure	Baseline	4 cmH_2_O	*p*	7 cmH_2_O	*p*	10 cmH_2_O	*p*	14 cmH_2_O	*p*	17 cmH_2_O	*p*
GTV											
Volume (cm^3^)	77.3 ± 48.4	77.4 ± 47.9	0.85	76.7 ± 49.2	0.74	73.8 ± 44.8	0.37	75.5 ± 44.3	0.94	69.1 ± 39.8	0.69
CTV											
Volume (cm^3^)	161.7 ± 78.3	162.9 ± 77.9	0.57	157.6 ± 81.6	0.32	156.3 ± 70.6	0.15	158.7 ± 70.6	0.11	151.4 ± 66.5	0.95
PTV											
Volume (cm^3^)	292.3 ± 129.0	286.9 ± 137.2	0.47	291.0 ± 127.1	0.72	284.1 ± 118.3	0.12	287.8 ± 118.3	0.03 *	284.3 ± 114.7	0.79
Lung											
Volume (cm^3^)	3318.0 ± 742.4	3575.4 ± 762.1	<0.01 *	3840.1 ± 764.5	<0.01 *	4148.9 ± 820.5	<0.01 *	44529.5 ± 905.9	<0.01 *	4883.2 ± 799.4	<0.01 *
D_mean_ (cGy)	1573.5 ± 314.0	1545.9 ± 327.7	0.08	1495.3 ± 330.8	<0.01 *	1455.9 ± 334.6	<0.01 *	1392.8 ± 304.4	<0.01 *	1443.4 ± 268.7	<0.01 *
V5 (%)	65.1 ± 13.7	64.0 ± 13.6	0.07	62.7 ± 13.1	<0.01 *	61.1 ± 13.5	<0.01 *	58.6 ± 13.2	<0.01 *	59.6 ± 12.3	<0.01 *
V10 (%)	50.7 ± 13.3	49.5 ± 13.4	0.03 *	48.2 ± 13.2	<0.01 *	47.1 ± 13.1	<0.01 *	44.8 ± 12.7	<0.01 *	46.8 ± 11.2	<0.01 *
V15 (%)	38.5 ± 10.7	37.5 ± 10.4	0.10	36.0 ± 10.2	<0.01 *	35.4 ± 10.1	<0.01 *	33.8 ± 9.6	<0.01 *	34.9 ± 8.0	<0.01 *
V20 (%)	28.1 ± 7.4	27.7 ± 7.5	0.24	26.6 ± 7.6	<0.01 *	26.0 ± 7.4	<0.01 *	25.1 ± 6.9	<0.01 *	26.0 ± 5.6	<0.01 *
Heart											
Volume (cm^3^)	725.6 ± 155.4	719.3 ± 162.0	0.63	702.6 ± 167.5	0.06	683.8 ± 134.5	0.05	679.0 ± 109.3	<0.01 *	622.8 ± 102.4	<0.01 *
D_mean_ (cGy)	1277.4 ± 601.1	1174.7 ± 566.5	0.27	1238.1 ± 612.1	0.14	1243.1 ± 607.7	0.13	1212.2 ± 653.4	0.02 *	1139.6 ± 651.9	0.01 *
V5 (%)	55.5 ± 25.8	54.0 ± 26.4	0.15	53.5 ± 27.2	0.08	53.8 ± 27.4	0.07	52.1 ± 27.8	<0.01 *	49.7 ± 25.8	<0.01 *
V50 (%)	3.6 ± 2.6	3.6 ± 2.5	0.88	3.3 ± 2.4	0.28	3.3 ± 2.3	0.42	3.1 ± 2.4	0.33	3.1 ± 2.4	0.50

(Mean ± standard deviation), statistically significant, *. Abbreviations: GTV, gloss target volume; CTV, clinical target volume; PTV, planning target volume, D_mean_, mean dose.

## Data Availability

The data presented in this study are available upon request from the corresponding author.

## Supplementary Materials

The following supporting information can be downloaded at: https://www.mdpi.com/article/10.3390/cancers14174308/s1, Figure S1: Lung volume and dose parameter change between interpressure; (A1) volume, (A2) Dmean, (A3) V5, (A4) V10, (A5) V15, and (A6) V20; Figure S2: Heart dose parameter change between interpressure; (B1) D_mean_, (B2) V5, and (B3) V50; Table S1: Dose constraints for the organs at risk.
