# Supplementary material for: Efficacy and Optimal Pressure of Continuous Positive Airway Pressure in Intensity-Modulated Radiotherapy for Locally Advanced Lung Cancer
Source: Cancers (Basel). 2022 Sep 2;14(17):4308. doi: 10.3390/cancers14174308 (PMC9454671; doi:10.3390/cancers14174308)
Supplement: Supplementary file 1 [file cancers-14-04308-s001.zip › cancers-1858751-supplementary.pdf]

Table S1. Dose constraints for the organs at risk

Organ	Dose constraints
Lung	D_{mean} <20 Gy
	V10 <40%
	V20 <35%
Heart	D_{mean} <35 Gy
	V30 <30 %
	V45 <35 %
Esophagus	V45 <33 %
Spinal cord	D_{max} <45 Gy

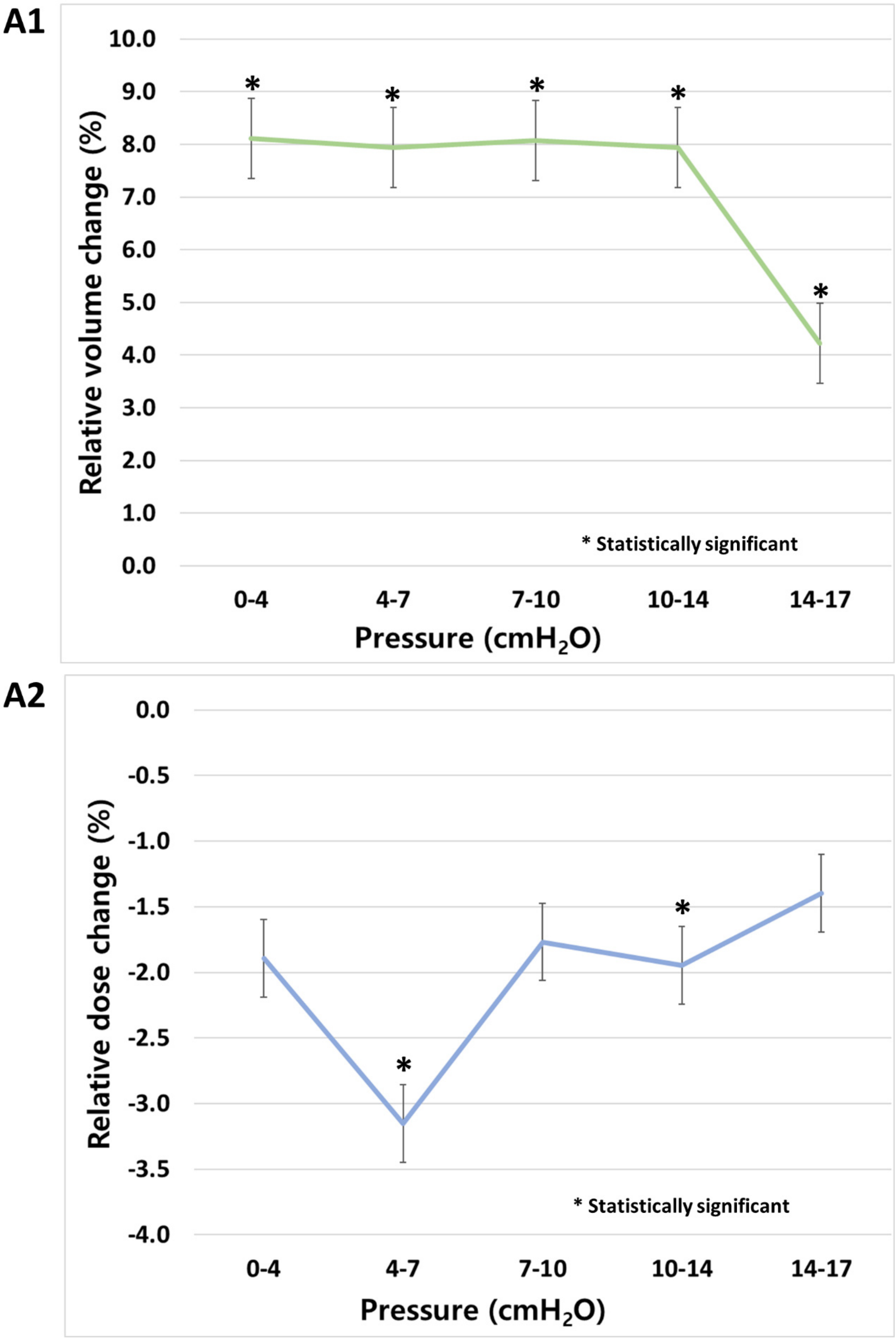
A1
10.0
9.0
8.0
7.0
6.0
5.0
4.0
3.0
2.0
1.0
0.0
Relative volume change (%)
* Statistically significant
0-4
4-7
7-10
10-14
14-17
Pressure (cmH2O)
A2
0.0
-0.5
-1.0
-1.5
-2.0
-2.5
-3.0
-3.5
-4.0
Relative dose change (%)
* Statistically significant
0-4
4-7
7-10
10-14
14-17
Pressure (cmH2O)

A3

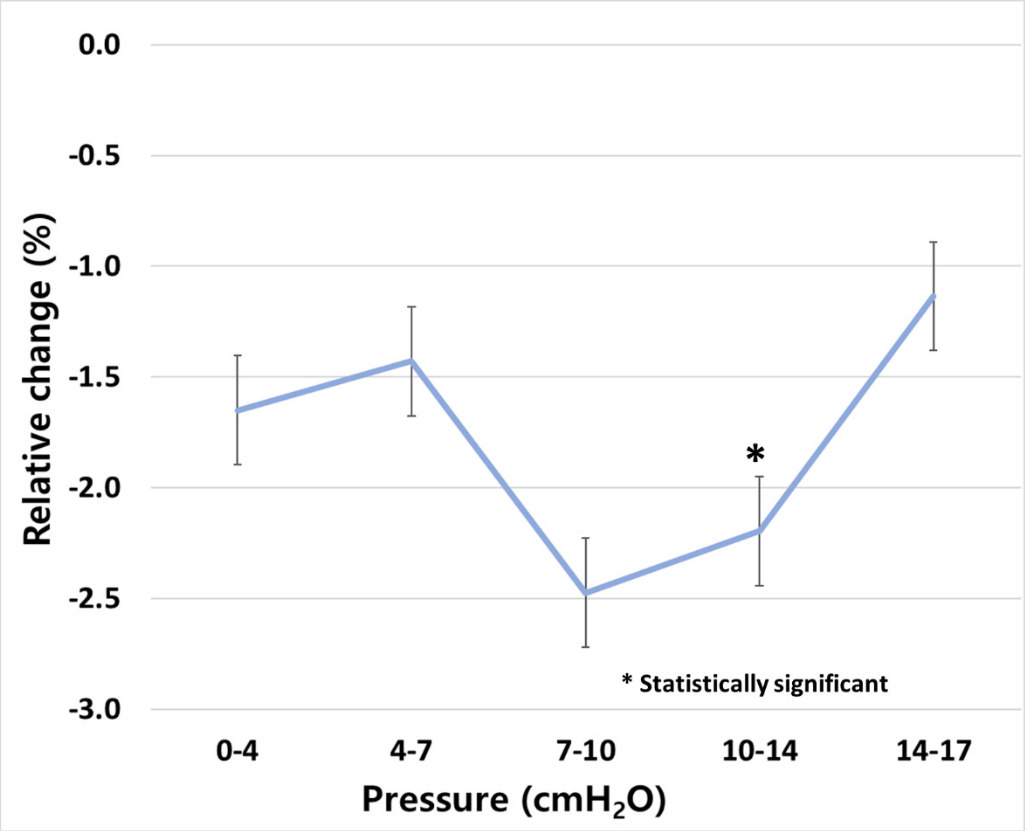
0.0
-0.5
-1.0
-1.5
-2.0
-2.5
-3.0
Relative change (%)
*
* Statistically significant
0-4
4-7
7-10
10-14
14-17
Pressure (cmH2O)

A4

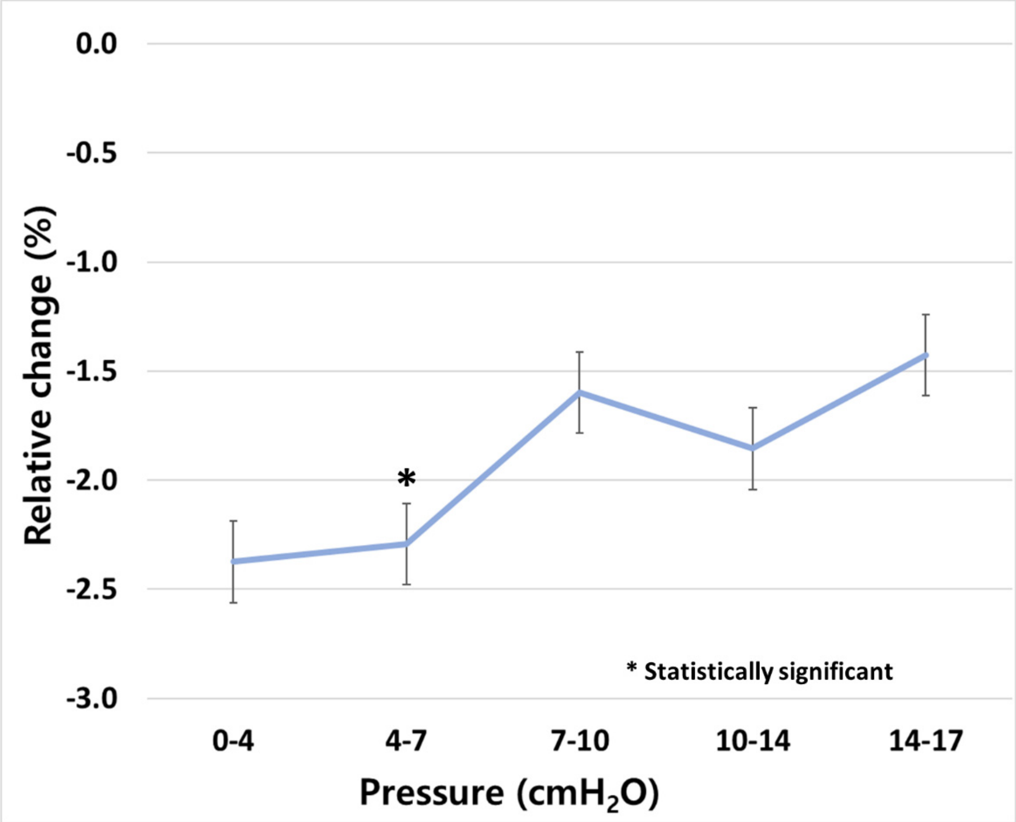
0.0
-0.5
-1.0
-1.5
-2.0
-2.5
-3.0
Relative change (%)
*
* Statistically significant
0-4
4-7
7-10
10-14
14-17
Pressure (cmH2O)

A5

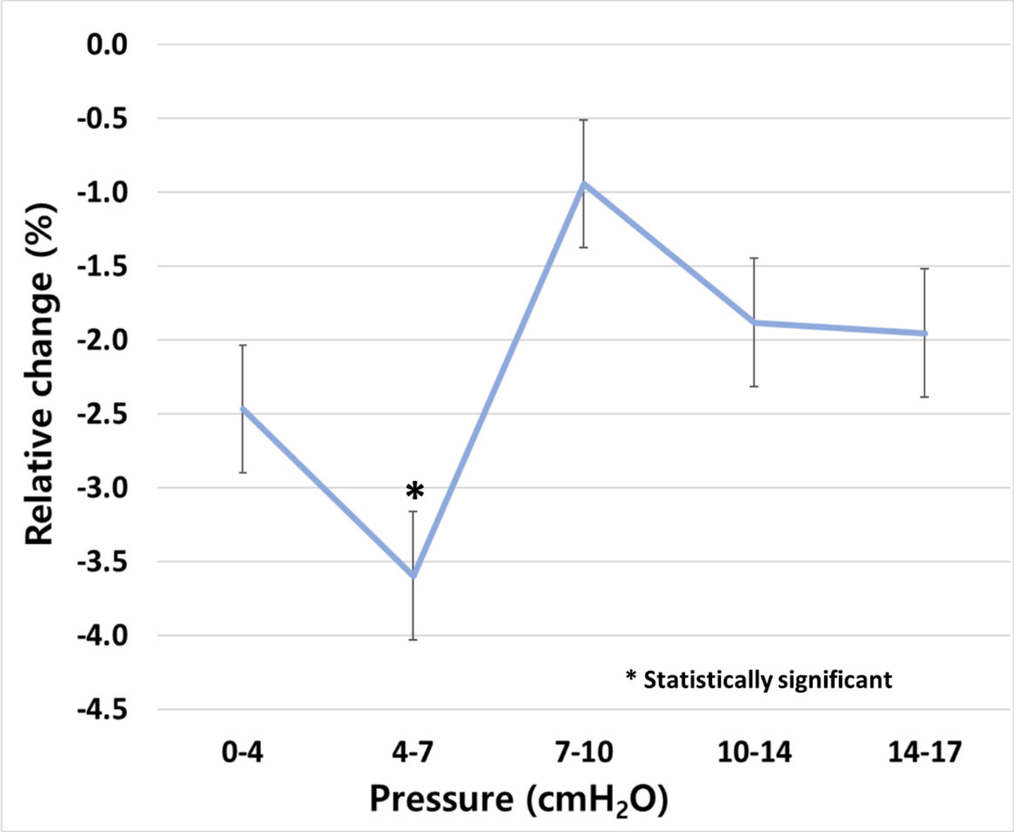

A6

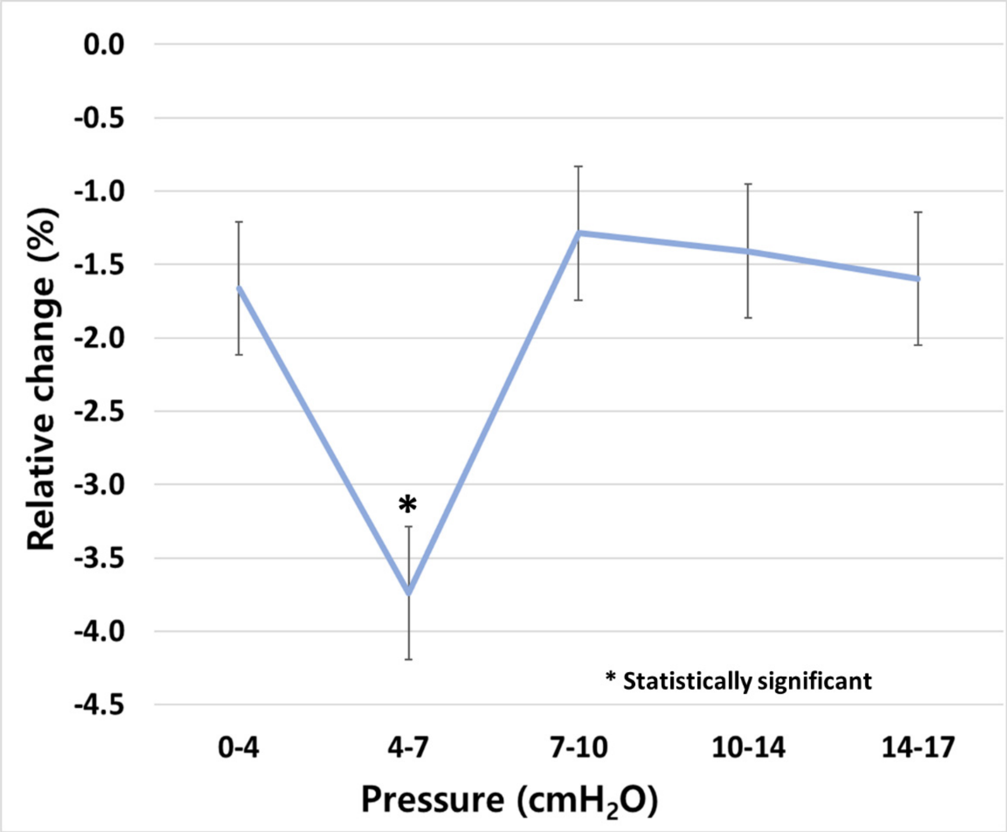

Figure S1. Lung volume and dose parameter change between interpressure; (1) volume, (2) D_{mean}, (3) V5, (4) V10, (5) V15, and (6) V20.

B1

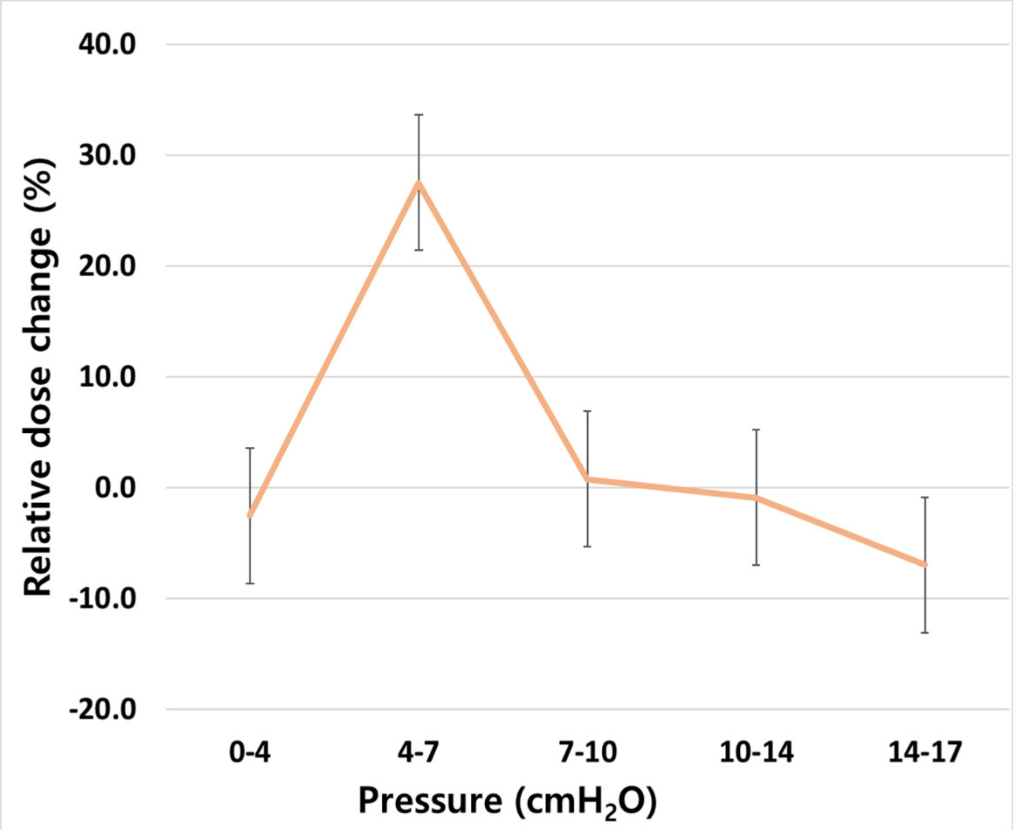
40.0
30.0
20.0
10.0
0.0
-10.0
-20.0
Relative dose change (%)
0-4
4-7
7-10
10-14
14-17
Pressure (cmH$_2$O)

B2

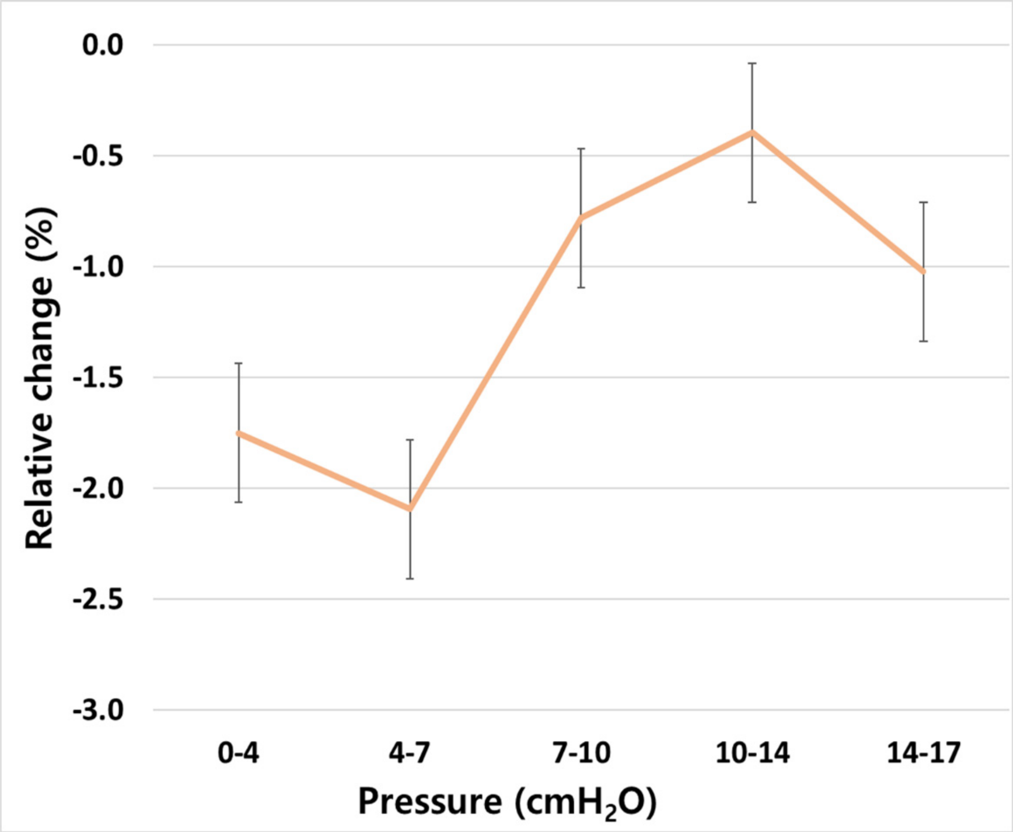
0.0
-0.5
-1.0
-1.5
-2.0
-2.5
-3.0
Relative change (%)
0-4
4-7
7-10
10-14
14-17
Pressure (cmH$_2$O)

B3

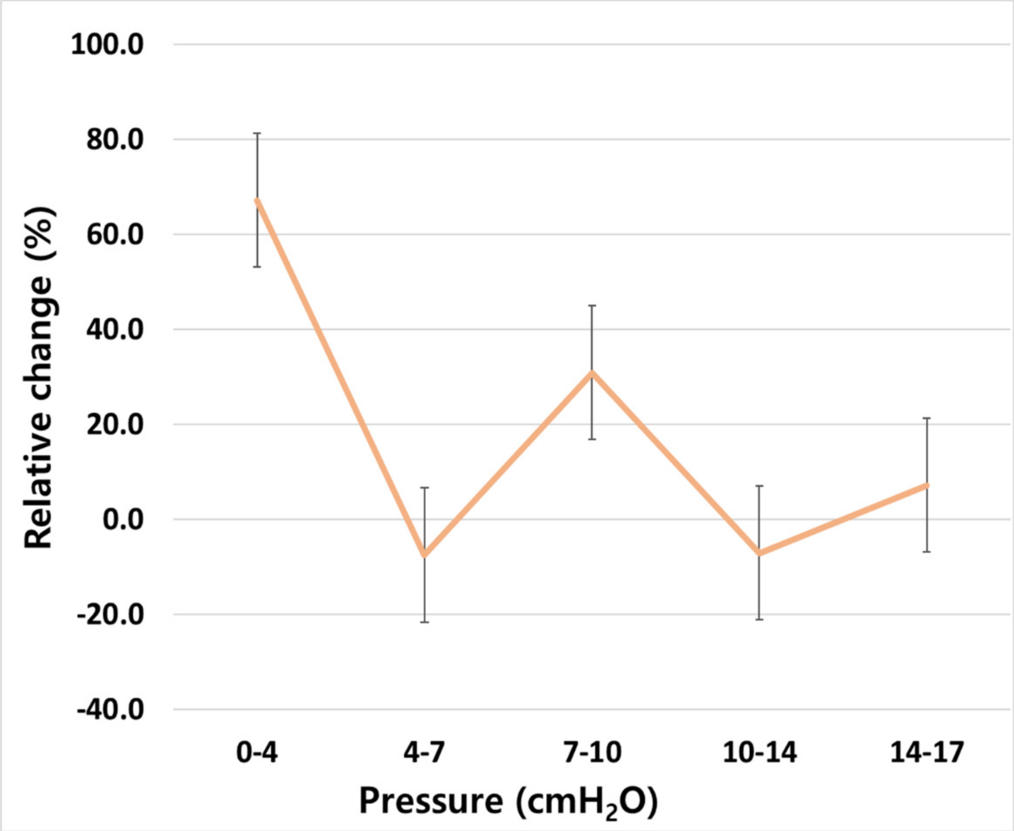

Figure S2. Heart dose parameter change between interpressure; (1) D_{mean}, (2) V5, and (3) V50.
